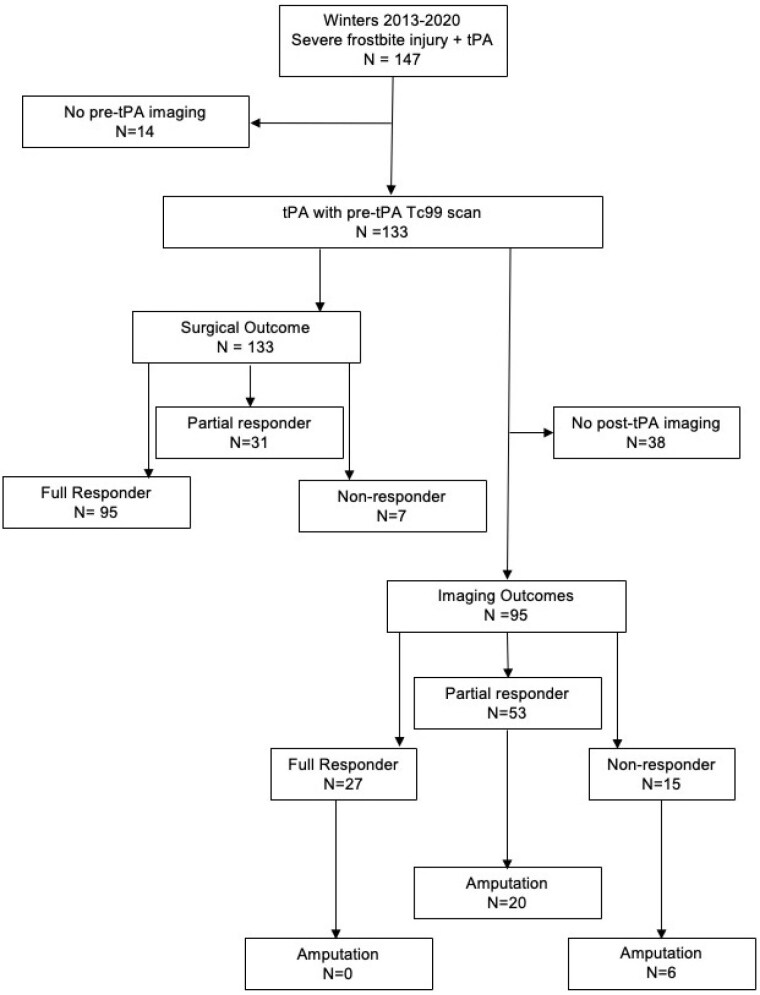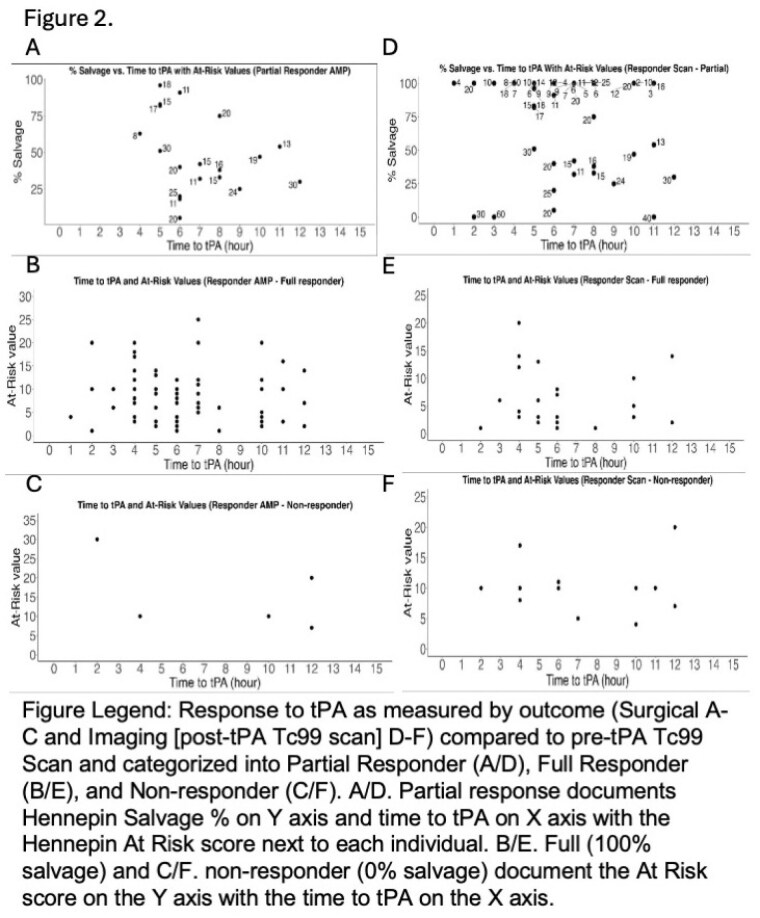# 90 Individual and Injury Characteristics of Response to Thrombolytic Therapy Following Severe Frostbite Injury

**DOI:** 10.1093/jbcr/iraf019.090

**Published:** 2025-04-01

**Authors:** Rachel Nygaard, Emily Colonna, Rediat Tilahun, Charly Vang, Alexandra Lacey, Kyle Schmitz, Derek Lumbard

**Affiliations:** Hennepin Healthcare; Hennepin Healthcare; Hennepin Healthcare Research Institute; Hennepin Healthcare Research Institute; Regions Hospital Burn Center; Hennepin Healthcare; Hennepin Healthcare

## Abstract

**Introduction:**

Approximately 30% of severe frostbite injuries result in amputation. Thrombolytic therapy (tPA) is used to reduce tissue loss following severe frostbite injury. This study evaluates factors impacting the tPA effectiveness using post-tPA perfusion imaging and amputation level as outcome measures. We hypothesize that stratifying tPA-treated patients into full, partial, and non-responders will allow for a more nuanced assessment of treatment efficacy.

**Methods:**

We reviewed a prospectively maintained frostbite database (2013–2020) for patients with post-rewarming perfusion deficits measured by Tc99 scan who were treated with IV tPA. Descriptive statistics and multinomial logistic regression assessed differences in responder categories (full, partial, non-responders), adjusting for time to tPA, pre-treatment tissue perfusion deficits, and infection.

**Results:**

Of 133 patients, 71.4% were full responders, 23.3% partial responders, and 5.3% non-responders for surgical outcome (Fig 1). The median time to tPA was 5.5 hours (range 3.5-14 for full responder); 7 hours (range 1.5-14 for partial responder); and 10 hours (range 1-10 for non-responder). Full responders had significantly smaller initial perfusion deficits than partial and non-responders (Fig 2 A-C). There were no significant differences in psychosocial or comorbid factors observed across responder groups. Time to tPA, amount of tissue with post-rewarming perfusion deficit, and cellulitis or infection significantly increased the risk of non-response relative to amputation in multinominal regression.

Since cellulitis/infection significantly impacts final amputation level, we used post-tPA imaging as an outcome measure less impacted by confounding factors. Imaging outcomes were available for 95 patients, 28.4% full responders, 55.8% partial responders, and 15.8% non-responders (Fig 1). Similar findings were observed regarding time to tPA and tissue impacted influencing response groups (Fig 2 D-F). Interestingly, significantly fewer imaging non-responders had a history of alcohol abuse. Full responders for imaging outcome corresponded with surgical outcomes and had no amputations, while 37.7% of partial responders and 40% of non-responders on imaging outcome had amputations (Fig 1). Of partial and non-responders for imaging outcomes that had amputations, all but 1 had predicted amputation needed on discharge planning.

**Conclusions:**

This study is the largest to evaluate tPA outcomes in severe frostbite injured patients, provides new insight into tPA responses, and offers a novel assessment of tPA treatment efficacy. These findings underscore the importance of timely tPA administration and demonstrate benefits for patients treated outside the standard tPA treatment windows.

**Applicability of Research to Practice:**

Early administration of tPA, within 5–7 hours of warming, was associated with the best outcomes, however tPA response was observed in a wide range of treatment times.

**Funding for the Study:**

N/A